# Mesenchymal Stem/Stromal Cell-Derived Extracellular Vesicles for Immunomodulation in Osteoarthritis

**DOI:** 10.3390/ijms27219922

**Published:** 2026-11-05

**Authors:** Michelle B. R. G. Ley, Marc C. Philippon, Ramy S. Labib, Miguel Fuentes-Chandia, Chao Liu, Thomas M. Best, Rodrigo A. Somoza, Dimitrios Kouroupis

**Affiliations:** 1 Department of Orthopedics, Miller School of Medicine, University of Miami, Miami, FL 33136, USAmcp182@miami.edu (M.C.P.); rsl127@miami.edu (R.S.L.); txb440@med.miami.edu (T.M.B.); 2 Diabetes Research Institute & Cell Transplant Center, Miller School of Medicine, University of Miami, Miami, FL 33136, USA; 3 Department of Biology, Case Western Reserve University, Cleveland, OH 44106, USA; maf209@case.edu (M.F.-C.); ras286@case.edu (R.A.S.); 4 Center for the Multimodal Evaluation of Structural Tissues, Case Western Reserve University, Cleveland, OH 44106, USA; 5 Asclepii Inc., Cleveland, OH 44106, USA; 6 Department of Biomedical Engineering, Case Western Reserve University, Cleveland, OH 44106, USA; cxl942@case.edu

**Keywords:** osteoarthritis, mesenchymal stem/stromal cells, extracellular vesicles, immunomodulation, synovial macrophages, immune–stromal crosstalk, synovitis, cell-free therapy, clinical translation

## Abstract

Osteoarthritis (OA) is a heterogeneous disorder affecting multiple joint tissues, in which structural degeneration may be accompanied by varying degrees of synovial inflammation and immune–stromal dysregulation. Mesenchymal stem/stromal cells (MSCs) have demonstrated immunomodulatory and tissue-protective properties, increasingly linked to paracrine signaling. Comparative studies suggest that extracellular vesicles (EVs) make an important, but not exclusive, contribution to MSC paracrine activity, with soluble secretome components providing potentially complementary effects. Drawing on literature identified primarily through PubMed and Scopus and clinical trial records from ClinicalTrials.gov (updated through September 2026), this narrative review examines the immunomodulatory role of MSC-derived EVs (MSC-EVs) in OA, with emphasis on their interactions with the joint immune microenvironment and their contribution to tissue protection. Across experimental OA models, MSC-EVs have been reported to reduce synovitis, cartilage degeneration, subchondral bone alterations, and pain-related outcomes, although responses vary with MSC source, EV composition, engineering strategies, disease context, and treatment regimen. Mechanistically, the most consistent evidence supports modulation of macrophage-associated inflammatory responses, whereas effects on adaptive immune populations remain less established and are largely derived from non-OA models. Clinical evidence remains limited and currently provides mainly preliminary information on feasibility and short-term tolerability, with insufficient evidence to establish therapeutic efficacy. Overall, MSC-EVs represent a potential cell-free immunomodulatory approach for OA, but standardized manufacturing, product characterization, dosing, and rigorously controlled clinical trials are required to define their therapeutic value.

## 1. Introduction

Despite decades of research, osteoarthritis (OA) remains a leading cause of chronic pain and disability, with no approved disease-modifying pharmacological therapy capable of reliably preventing or reversing disease progression [1,2]. In 2021, approximately 46.6 million new OA cases were estimated worldwide, corresponding to an age-standardized incidence rate of 535 per 100,000 population [3]. This therapeutic gap reflects a fundamental challenge: OA is not driven by a single pathological process, but by interconnected mechanical, metabolic, inflammatory, and degenerative mechanisms that vary across patients and disease stages [4,5]. Consequently, effective disease modification may require more than protecting individual joint tissues; it may depend on restoring the biological interactions that sustain joint homeostasis.

Mesenchymal stem/stromal cells (MSCs) have emerged as candidates for addressing this complexity through their immunomodulatory and trophic activities. Although initially investigated for their differentiation and tissue-regenerative potential, their therapeutic effects are now understood to depend substantially on paracrine signaling [6,7]. Among the components of the MSC secretome, MSC-derived extracellular vesicles (MSC-EVs) have gained attention for their capacity to deliver regulatory molecules and modulate inflammatory signaling in recipient cells [8,9]. As cell-free therapeutic candidates, MSC-EVs offer opportunities for controlled formulation and engineering without requiring viable-cell administration, although their biological activity may not fully reproduce that of the broader MSC secretome.

Across experimental OA models, MSC-EVs have been reported to attenuate synovial inflammation and cartilage degeneration, supporting their therapeutic potential [10,11,12]. However, the contribution of specific immunomodulatory mechanisms to joint protection remains incompletely established, while heterogeneity in extracellular vesicle (EV) preparations and experimental designs complicates comparisons across studies [13,14]. Clinical investigations remain at an early stage, with insufficient evidence to establish the therapeutic efficacy of MSC-EVs in OA [15,16,17].

Accordingly, this review critically evaluates evidence for MSC-EV-mediated immunomodulation in OA, linking innate and adaptive immune responses to structural and functional outcomes while distinguishing OA-specific evidence from mechanisms extrapolated from other inflammatory conditions. It further examines how MSC source, EV composition, engineering, delivery, and manufacturing influence therapeutic activity, identifying key priorities for mechanistic validation, standardization, and clinical translation.

## 2. MSC-EVs in Osteoarthritis: Biological Rationale, Mechanisms, and Translational Evidence

### 2.1. Pathophysiology and Clinical Landscape of Osteoarthritis

Osteoarthritis (OA) is a prevalent, heterogeneous whole-joint disorder characterized by interconnected pathological changes in articular cartilage, synovium, subchondral bone, menisci, infrapatellar fat pad (IFP), and periarticular tissues [18,19]. Rather than progressing independently, alterations across these compartments are linked through mechanical and biochemical crosstalk, creating reciprocal relationships between tissue degeneration, inflammatory signaling, and pain [20,21]. The relative involvement of individual joint compartments varies among patients and disease stages, contributing to diverse clinical presentations and complicating the identification of therapeutic targets capable of consistently modifying disease progression [4].

OA pathogenesis reflects interactions among genetic susceptibility, aging, abnormal mechanical loading, and systemic metabolic factors [1,22]. Genetic studies have identified numerous susceptibility loci associated with cartilage homeostasis, skeletal development, and other disease-related processes, although their contributions are influenced by environmental and lifestyle factors [23,24,25]. Aging further disrupts joint homeostasis through cellular senescence and chronic low-grade inflammation, commonly termed inflammaging, while metabolic dysfunction and excessive mechanical stress promote oxidative injury, extracellular matrix (ECM) degradation, and inflammatory responses [26,27,28]. The relative contributions of these processes vary across individuals and joint compartments, creating heterogeneous conditions for tissue degeneration and repair.

Although OA is not classified as a classical inflammatory arthritis, inflammation contributes to disease pathology in a substantial proportion of patients, with its intensity and distribution varying across disease stages and joint compartments [29]. The synovium represents an important site of immune–stromal crosstalk, with synovitis detectable throughout the disease course, including early stages, and associated with symptomatic manifestations and structural progression [5,30]. Within the OA joint, damaged cartilage and other tissues release damage-associated molecular patterns (DAMPs), which activate innate immune pathways and stimulate inflammatory responses in synovial macrophages, fibroblasts, and other joint-resident cells [31,32]. These interactions promote the production of inflammatory mediators, including interleukin (IL)-1β, tumor necrosis factor (TNF)-α, IL-6, and chemokines, contributing to an environment that favors matrix catabolism and impaired tissue repair [31,32]. In turn, inflammatory signals influence chondrocyte function and subchondral bone remodeling, reinforcing pathological communication among joint compartments [20,21,31].

Within this inflammatory network, synovial macrophages participate in immune surveillance, tissue homeostasis, and responses to joint injury, while macrophage-associated inflammatory signaling can contribute to synovitis, cartilage degradation, and pain-related responses [32,33]. Although experimental studies frequently characterize macrophages according to pro-inflammatory M1-like and anti-inflammatory or reparative M2-like phenotypes, this binary framework does not adequately represent the functional diversity of macrophage populations within OA tissues [34]. Macrophage states are heterogeneous and dynamic, reflecting interactions among tissue origin, disease stage, local metabolic and inflammatory conditions, and communication with neighboring stromal cells [19,34]. Consequently, therapeutic macrophage regulation should be understood as the modulation of context-dependent functional states rather than a simple transition between two fixed phenotypes.

Adaptive immune populations may further contribute to the OA inflammatory environment. T- and B-cell subsets have been identified in OA synovial tissues, where their interactions with innate immune and stromal populations may influence local inflammatory activity [29,35]. Nevertheless, their functional contributions remain less clearly defined than those of innate immune pathways, and the relevance of particular lymphocyte subsets likely varies with disease context. More broadly, differences in inflammatory activity, metabolic dysfunction, structural pathology, and pain-related mechanisms have led to growing interest in OA phenotyping as a means of understanding disease heterogeneity and potentially guiding therapeutic selection [4,36,37,38].

Current OA management remains directed primarily toward symptom control and functional improvement through non-pharmacological strategies, including exercise and weight management, together with pharmacological approaches such as nonsteroidal anti-inflammatory drugs (NSAIDs) and selected intra-articular (IA) interventions [39,40]. Although these strategies can provide symptomatic benefit, they have not reliably altered the underlying trajectory of structural disease, and no disease-modifying osteoarthritis drug (DMOAD) is currently approved [2]. Moreover, the heterogeneity of inflammatory activity and its relationship with structural degeneration suggests that interventions targeting immune regulation may not produce equivalent therapeutic benefits across all OA phenotypes [29,36,37,38].

Together, these observations establish OA as a disorder characterized by interconnected immune, stromal, and structural disturbances rather than isolated cartilage degeneration. This complexity provides a rationale for investigating therapeutic approaches capable of modulating inflammatory signaling while supporting joint tissue homeostasis. However, determining whether such interventions can achieve clinically meaningful disease modification requires identification of relevant cellular targets, establishment of causal relationships between immune regulation and joint protection, and clarification of the disease contexts in which these mechanisms are most likely to be beneficial.

### 2.2. MSC Paracrine Immunomodulation

MSCs exert many of their therapeutic effects through paracrine signaling rather than long-term engraftment or direct differentiation [41,42]. This activity is mediated by a dynamic secretome comprising cytokines, chemokines, growth factors, lipid mediators, metabolites, ECM-modifying molecules, and EVs [43,44,45]. Secretome composition is strongly influenced by the cellular microenvironment, allowing MSCs to adapt their immunoregulatory activity in response to inflammatory and tissue-derived signals [42,46].

Inflammatory cytokines such as interferon (IFN)-γ, TNF-α, and IL-1β can functionally “license” MSCs, altering the expression and release of immunomodulatory mediators and enhancing their capacity to regulate immune-cell activity [47,48,49,50,51]. Hypoxia, oxidative stress, mechanical cues, cell density, tissue source, and culture conditions may further modify MSC paracrine function [52,53]. Consequently, the immunomodulatory activity of MSCs cannot be attributed to a single constitutively expressed factor but instead reflects coordinated and stimulus-dependent paracrine responses.

**Figure 1:**
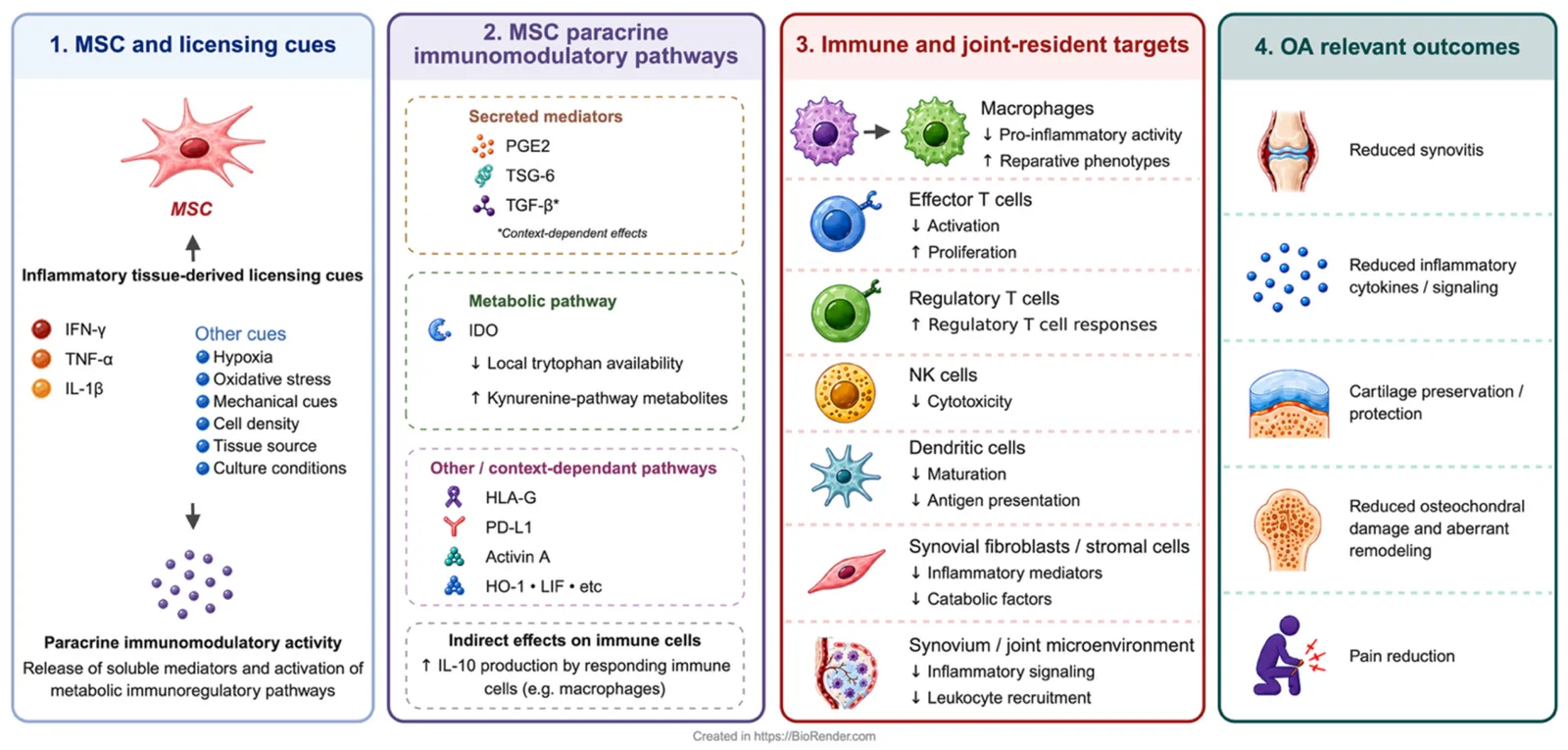
MSC paracrine immunomodulation in osteoarthritis. Inflammatory and tissue-derived signals within the osteoarthritic joint, including IFN-γ, TNF-α, IL-1β, hypoxia, oxidative stress, and mechanical cues, can functionally license mesenchymal stem/stromal cells (MSCs) and modify their paracrine activity. MSC immunomodulation involves secreted mediators, including prostaglandin E2 (PGE2), tumor necrosis factor-stimulated gene 6 protein (TSG-6), and transforming growth factor-β (TGF-β), as well as metabolic pathways such as indoleamine 2,3-dioxygenase (IDO)-mediated tryptophan catabolism. Additional context-dependent pathways include HLA-G, PD-L1, activin A, heme oxygenase-1 (HO-1), and leukemia inhibitory factor (LIF). MSC-derived signals can also indirectly promote IL-10 production by responding to immune cells. Collectively, these pathways modulate macrophages, effector and regulatory T cells, NK cells, DCs, synovial mesenchymal stem/stromal cells (SMSCs), and the joint microenvironment, contributing to reduced inflammatory signaling and synovitis, preservation of cartilage and osteochondral homeostasis, and potentially reduced pain. The relative contribution of individual pathways is context-dependent, and the strength of OA-specific mechanistic evidence varies among mediators.

Among the best-characterized pathways involved in MSC immunoregulation are the soluble mediators prostaglandin E2 (PGE2), tumor necrosis factor-stimulated gene 6 protein (TSG-6), and transforming growth factor-β (TGF-β), together with metabolic pathways such as indoleamine 2,3-dioxygenase (IDO) [54,55,56]. These mechanisms can influence both innate and adaptive immunity by suppressing effector T-cell responses, modulating natural killer (NK)- and dendritic-cell (DC) activity, supporting regulatory T-cell responses, and promoting macrophage phenotypes associated with inflammation resolution and tissue repair [57,58,59]. The principal MSC paracrine immunomodulatory pathways and their OA-relevant cellular targets and outcomes are summarized in Figure 1.

PGE2 is a lipid mediator generated through cyclooxygenase-dependent pathways and is increased in MSCs following inflammatory stimulation. It is among the most established mediators of MSC-dependent immune regulation and can suppress T-cell, NK-cell, and dendritic-cell activity while modulating macrophage inflammatory responses [60,61,62,63,64,65,66,67]. Importantly, PGE2 can also promote IL-10 production by responding to macrophages and other immune cells. Thus, increased IL-10 signaling following MSC treatment may result partly from indirect reprogramming of host immune cells rather than exclusively from direct MSC secretion. This distinction is relevant when interpreting OA studies using IL-10-overexpressing or IL-10-enriched MSCs, which have demonstrated reduced synovial inflammation, inflammatory cytokine expression, and structural disease progression [68,69] but do not establish endogenous MSC-derived IL-10 as the principal mediator of these effects.

TSG-6 is another inflammation-responsive mediator with particular relevance to OA. MSCs can increase TSG-6 secretion following inflammatory stimulation, and TSG-6 has anti-inflammatory and matrix-protective properties [70,71,72]. In OA tissues, TNFAIP6/TSG-6 expression has been associated with chondrocyte phenotypes exhibiting lower MMP13 expression, while recombinant TSG-6 reduced cartilage degradation in human OA explants and attenuated cartilage damage and tactile allodynia in experimental OA [73,74,75,76]. TSG-6 may additionally regulate protease-dependent ECM remodeling through interactions with inter-α-inhibitor [77]. These findings support the biological relevance of TSG-6 in OA, although recombinant-protein studies establish TSG-6 activity rather than its specific contribution to MSC-mediated therapeutic effects.

TGF-β also contributes to MSC-mediated immune regulation and tissue homeostasis. Depending on cellular and tissue context, TGF-β can support regulatory T-cell differentiation, modulate inflammatory signaling, and promote chondrogenic matrix synthesis [78,79,80]. Its effects in OA are nevertheless context-dependent: appropriately regulated TGF-β signaling contributes to cartilage homeostasis and repair, whereas excessive or spatially dysregulated signaling may promote fibrosis, osteophyte formation, and pathological osteochondral remodeling [81,82,83]. TGF-β should therefore be considered a pleiotropic component of MSC paracrine signaling rather than a uniformly protective OA mediator.

In contrast to secreted mediators such as PGE2, TSG-6, and TGF-β, indoleamine 2,3-dioxygenase (IDO) is primarily an intracellular immunoregulatory enzyme rather than a soluble secretome component. In human MSCs, IDO is strongly induced by inflammatory signals, particularly IFN-γ, and catalyzes tryptophan metabolism through the kynurenine pathway [84,85]. The resulting alterations in local tryptophan availability and accumulation of kynurenine-pathway metabolites can suppress T-cell proliferation and contribute to an immunoregulatory microenvironment [86,87]. IDO is therefore an established MSC immunoregulatory pathway, although its specific contribution to MSC-mediated OA effects remains to be defined [88,89].

Other molecules frequently measured in MSC secretome studies should be distinguished from direct therapeutic effectors. For example, IL-6 is an important OA-associated inflammatory cytokine, and reductions in IL-6 following MSC secretome treatment provide evidence of an anti-inflammatory response in stimulated chondrocytes and other joint cells [90,91]. However, such changes are better interpreted as downstream readouts of secretome activity than as evidence that IL-6 itself mediates MSC therapeutic effects. Other pathways, including HLA-G, PD-L1, activin A, heme oxygenase-1, and leukemia inhibitory factor, also contribute to MSC immunobiology [46,92], although their roles in OA are less well characterized.

Collectively, MSC paracrine immunomodulation reflects coordinated interactions among soluble, lipid, and metabolic pathways rather than the activity of a single dominant mediator. These mechanisms can regulate macrophage and lymphocyte responses, inflammatory signaling, synovial inflammation, and tissue homeostasis, although the strength of OA-specific mechanistic evidence varies among individual pathways and frequently derives from in vitro or non-OA models.

Soluble and metabolic mediators, however, represent only part of MSC paracrine activity. MSC-EVs provide a distinct mode of intercellular communication whose biological effects depend on cellular source, cargo composition, surface characteristics, recipient-cell uptake, and local biodistribution. The following section therefore examines MSC-EV biology and the mechanisms through which these vesicles modulate the OA immune microenvironment.

### 2.3. MSC-EV Immunomodulation and Joint Protection in Osteoarthritis

According to the Minimal Information for Studies of Extracellular Vesicles 2023 (MISEV2023) guidelines, EVs are particles released from cells, delimited by a lipid bilayer, and unable to replicate independently [13]. EVs can carry proteins, lipids, nucleic acids, metabolites, and other bioactive components, with their molecular composition influenced by the identity and physiological state of the parent cell [8,13]. As a distinct component of MSC paracrine signaling, MSC-EVs can interact with recipient cells and modulate inflammatory signaling, immune-cell behavior, and processes associated with cartilage homeostasis and tissue remodeling [93,94,95].

Importantly, MSC-EVs exhibit molecular cargo profiles that do not simply mirror those of their parental cells. Paired miRNA profiling of human bone marrow-derived MSCs (BMSCs) and their derived EVs (BMSC-EVs) generated under xeno-free conditions identified distinct cellular and EV-associated signatures, with 42 miRNAs differentially represented between the two compartments. These findings are consistent with selective cargo packaging, while pathway analysis linked the differentially represented miRNAs to biological processes including immune regulation, proliferation, and differentiation [96].

#### 2.3.1. Innate Immune Modulation in the OA Joint

Among the immunomodulatory effects of MSC-EVs investigated in OA, regulation of synovial macrophages is supported by the most direct in vivo evidence. Across several chemically and surgically induced OA models, IA MSC-EV administration has reduced macrophage-associated inflammatory activity together with synovitis, inflammatory cytokine expression, and cartilage degeneration [10,11,12,97,98,99].

MSC-EVs from different tissue sources appear capable of producing this immunoregulatory response. BMSC-EVs in a rat model of modified Hulth surgery-induced OA reduced synovial macrophage infiltration and inflammatory cytokines while attenuating cartilage damage and osteophyte formation [12]. Umbilical cord MSC-derived EVs (UC-MSC-EVs) similarly reduced TNF-α, IL-1β, and IL-6 while increasing IL-10 and macrophage markers associated with reparative activity in rats with ACLT-induced OA [97]. Adipose-derived MSC-EVs (AD-MSC-EVs) reduced synovial and IFP inflammation and cartilage degeneration in MIA-induced OA in rats, with single-cell transcriptomic analysis identifying changes in macrophage states together with altered inflammatory and joint-homeostatic signaling [10]. IFP-MSC-EVs also demonstrated immunomodulatory activity by suppressing inflammatory macrophage responses, accompanied by reduced synovitis and IFP fibrosis in MIA-induced OA in rats with an acute synovial/IFP inflammatory phenotype [98]; in ACLT-induced OA in mice, IFP-MSC-EVs decreased cytokine expression, cartilage degeneration, osteophyte formation, and subchondral bone abnormalities [11]. Collectively, these findings indicate that MSC-EV-mediated immunoregulation involves both macrophage responses and broader changes within the synovial immune–stromal environment.

Mechanistic studies indicate that this activity is highly dependent on EV cargo and parental-cell conditioning. Synovial MSC-derived EVs (SMSC-EVs) carrying miR-3614-5p targeted ANXA2 and suppressed TLR4/MyD88/NF-κB signaling, thereby reducing inflammatory macrophage activity and cartilage destruction in a study incorporating ACLT-induced OA in rats [99]. IL-6-primed MSC-EVs altered macrophage metabolism through the mt-ND3/NADH-CoQ axis and produced greater reductions in synovitis and cartilage erosion than unprimed EVs in rats with combined ACLT/DMM-induced OA [100]. BMSC-EVs have also been reported to regulate macrophage responses through the PINK1/Parkin pathway in a study using ACLT combined with medial meniscus resection in rats [101], while BMP-7 modification enhanced the immunomodulatory and chondroprotective activity of SMSC-EVs in MIA-induced OA in rats [102]. These findings demonstrate that parental-cell conditioning and EV cargo can modify MSC-EV immunoregulatory activity.

#### 2.3.2. Adaptive Immune Modulation: Emerging Evidence

Compared with macrophage regulation, direct evidence for MSC-EV-mediated modulation of adaptive immunity in OA remains limited. Both T- and B-cell populations are present in OA synovial tissues, where they may contribute to inflammatory or regulatory immune responses [103,104,105].

Among adaptive immune populations, T cells have received greater mechanistic attention in MSC-EV studies. Evidence from non-OA inflammatory and autoimmune models indicates that MSC-EVs can suppress CD4+ T-cell activation and proliferation, reduce pro-inflammatory Th1- and Th17-associated responses, and, in some settings, promote regulatory or tolerogenic T-cell phenotypes [106,107,108,109,110]. These effects involve multiple mechanisms. Membrane-bound TGF-β has been identified on MSC-derived microvesicles [110], while TGF-β detected in endometrial MSC-EV preparations contributes to the suppression of CD4+ T-cell responses [109]. Separately, CD73 expressed on MSC-EVs catalyzes the conversion of AMP into immunosuppressive adenosine, a pathway implicated in the inhibition of activated T-cell proliferation [111]. Supporting this pathway in vivo, BMSC administration in a mouse model of human Th1-driven xenogeneic graft-versus-host disease has also been associated with increased circulating CD73+ EVs and adenosine-dependent suppression of Th1 responses [112]. The magnitude and nature of T-cell modulation vary among MSC-EV preparations and recipient immune environments, emphasizing the functional heterogeneity of EV-mediated immunomodulation [113].

B-cell modulation has been investigated less extensively. MSC-EVs have been shown to alter B-cell proliferation, differentiation, and antibody production. BMSC-EVs reduced activated B-cell proliferation and IgM production in cultured human B lymphocytes [114], while MSC-derived membrane vesicles have also been reported to suppress B-cell proliferation and differentiation in a CpG-stimulated peripheral blood mononuclear cell coculture system [115]. Conversely, other studies found that MSC-EVs did not fully reproduce the B-cell-regulatory effects of whole MSC-conditioned medium in comparative in vitro experiments, particularly induction of IL-10-producing regulatory B cells [116].

In vivo studies outside OA provide further evidence of adaptive immune regulation by MSC-EVs. In experimental lupus nephritis in lupus-prone MRL/lpr mice, UC-MSC-EVs reduced Th1/Th17-associated responses, increased regulatory T-cell populations, decreased plasma-cell abundance and anti-dsDNA IgG levels, and improved renal pathology [117]. In autoimmune dacryoadenitis in rabbits, UC-MSC-EVs increased regulatory T-cell responses and reduced inflammatory tissue damage, with miR-100-5p-dependent macrophage modulation contributing to these effects [118]. In collagen-induced inflammatory arthritis in mice, MSC-EVs reduced disease severity and were associated with fewer plasmablasts, increased Breg-like populations, and modulation of T-cell responses [119]. Together, these studies establish biological plausibility for MSC-EV-mediated regulation of both T- and B-cell responses but do not demonstrate that the same mechanisms drive therapeutic effects in OA.

#### 2.3.3. Preclinical Models and Joint-Level Outcomes

Preclinical evaluation of MSC-EVs in OA has relied predominantly on surgically and chemically induced rodent models. Surgical approaches include destabilization of the medial meniscus (DMM) and anterior cruciate ligament transection (ACLT), alone or in combination with meniscal injury, which induce joint instability and model features of post-traumatic OA. DMM generally produces more gradual structural deterioration, whereas ACLT and combined destabilization procedures generate greater instability and more rapid joint damage [120,121]. Chemically induced models include collagenase, which promotes ligamentous and matrix disruption with subsequent joint instability and inflammation, and monosodium iodoacetate (MIA), which induces rapid chondrocyte injury and is frequently used to evaluate structural degeneration and pain-related behavior [120,121]. These models differ in disease kinetics, inflammatory burden, and structural severity; consequently, therapeutic effects observed across models should not be considered directly equivalent.

Complementing animal models, human-based multicompartment Organ-on-Chip platforms offer opportunities to investigate OA-associated tissue crosstalk under controlled conditions. Vascularized microfluidic models incorporating cartilage, bone, synovial, and vascular compartments have been developed to investigate inflammatory responses and therapeutic interventions [122]. Similarly, vascularized osteochondral platforms have reproduced inflammation-associated endothelial and stromal invasion into cartilage [123]. Although MSC-EV efficacy has not been established in these models, they may provide valuable tools for future investigations of EV-mediated immunomodulation and joint tissue responses.

Despite this heterogeneity, MSC-EV administration has been associated with preservation of cartilage structure and chondrocyte homeostasis across different OA models, including DMM- and collagenase-induced OA in mice and ACLT-based or MIA-induced OA in rats [124,125,126,127,128,129,130]. Reported effects include maintenance or restoration of type II collagen and aggrecan, suppression of catabolic mediators such as MMP13 and ADAMTS5, and reductions in chondrocyte apoptosis or senescence [127,128,129,130,131,132]. Several studies have identified EV-associated molecular mechanisms contributing to these effects. IFP-MSC-EVs enriched in miR-100-5p inhibited mechanistic target of rapamycin (mTOR) signaling and enhanced autophagy, reducing cartilage degeneration and improving gait abnormalities in DMM-induced OA in mice [130]. In a mouse model of surgically induced OA involving medial collateral ligament and medial meniscus injury, MSC-EV-associated miR-3960 reduced chondrocyte injury through regulation of the PHLDA2/SDC1/Wnt/β-catenin axis [131], whereas the circHIPK3/miR-124-3p/MYH9 axis promoted chondrocyte proliferation and survival in a study incorporating collagenase-induced OA in mice [132]. Together, these findings indicate that MSC-EV-mediated cartilage protection involves regulation of both matrix homeostasis and chondrocyte survival.

Chondroprotective activity can also be influenced by parental-cell conditioning, EV cargo modification, and MSC source. Wogonin pretreatment of IFP-MSCs enhanced the cartilage-protective activity of their EVs in DMM-induced OA [133], while miR-140-5p overexpression in SMSCs generated EVs that preserved cartilage matrix and attenuated OA progression in rats with ACLT combined with medial collateral ligament and medial meniscus transection [134]. In a direct comparison of MSC sources, both induced pluripotent stem cell-derived MSC-EVs (iPSC-MSC-EVs) and SMSC-EVs exerted protective effects in collagenase-induced OA in mice, although iPSC-MSC-EVs produced greater cartilage preservation [135]. In MIA-induced OA in rats, CD10^High^IFP-MSC-EVs similarly reduced cartilage degeneration, preserved collagen composition, and maintained proteoglycan 4 (PRG4)-associated cartilage homeostasis [136]. Cartilage-targeted engineering has also been explored using AD-MSC-EVs carrying miR-199a-3p and modified with a chondrocyte-affinity peptide, which enhanced cartilage targeting and attenuated OA progression through regulation of the mTOR–autophagy pathway in studies involving combined DMM/ACLT-induced OA in rats and DMM-induced OA in mice; the engineered-EV experiments were conducted in the mouse model [137].

MSC-EV effects also extend beyond articular cartilage to the subchondral bone and broader osteochondral unit. In collagenase-induced OA in mice, BMSC-EVs preserved cartilage while reducing osteophyte formation and abnormal subchondral bone changes [126]. In an ACLT/DMM model in rats, BMSC-EVs attenuated cartilage destruction and abnormal subchondral bone remodeling, including restoration of trabecular bone volume, number, and connectivity [127]. IFP-MSC-EVs similarly reduced cartilage degeneration, osteophyte formation, and subchondral bone abnormalities in ACLT-induced OA in mice [11]. In MIA-induced OA in mice, dental pulp MSC-derived EVs (DP-MSC-EVs) improved subchondral bone architecture and reduced osteophyte formation by suppressing TRPV4-associated osteoclast activation [138]. These observations support a joint-level effect of MSC-EVs that extends beyond isolated cartilage preservation.

Therapeutic responses may also depend on the systemic disease environment. In a rat model combining high-fat-diet-induced metabolic dysfunction with surgically induced cartilage injury (groove model), MSC treatment was associated with greater joint degeneration and higher OARSI scores than vehicle treatment, whereas MSC-EV administration produced joint pathology comparable to controls and reduced systemic inflammatory biomarkers [139]. These findings suggest that metabolic and inflammatory status may influence responses to MSC-based interventions and should be considered when comparing therapeutic outcomes across OA phenotypes.

Vascular remodeling represents another component of osteochondral pathology, as pathological angiogenesis in subchondral bone can accompany abnormal bone remodeling and sensory nerve ingrowth. In ACLT-induced OA in mice, TGF-β1-modified BMSC-EVs reduced osteoclastogenesis and CD31^hi^Emcn^hi^ H-type vessel activity while preserving cartilage and subchondral bone microarchitecture [140]. These changes were accompanied by reduced OA-associated pain behavior, supporting a relationship among subchondral bone remodeling, pathological angiogenesis, and symptomatic outcomes. More broadly, pain and functional outcomes have been assessed less consistently than histological and molecular endpoints; however, improvements in gait or pain-related behavior have been reported in mouse ACLT- and DMM-induced OA and rat MIA-induced OA following treatment with IFP-MSC-EVs, AD-MSC-EVs, and modified BMSC-EVs [11,129,130,140]. Genetically modified IFP-MSC-EVs carrying the CGRP antagonist CGRP_8−37_ further reduced tactile and cold allodynia and joint-related pain in MIA-induced OA in rats with an acute synovial/IFP inflammatory phenotype while preserving cartilage structure, collagen organization, and PRG4-associated localization [141]. Because structural preservation does not necessarily predict symptomatic improvement, standardized functional and pain-related assessments will be important for evaluating translational relevance.

Limited within-study comparisons suggest that higher EV doses may enhance selected anti-inflammatory and pain-related responses, although improvements are not consistent across all endpoints [98,129]. Similarly, a recent network meta-analysis of rat OA models suggested greater therapeutic efficacy with higher EV doses, although most direct dose comparisons did not reach statistical significance [142]. However, cross-study comparisons are complicated by differences in OA model, EV preparations, treatment schedules, and dose quantification methods, including particle number, protein mass, and parental-cell equivalents. Although overt dose-related toxicity has not been established, limited systematic safety assessments prevent conclusions regarding an upper safe dose.

Overall, preclinical evidence indicates that MSC-EVs can exert joint-protective effects across diverse OA models, including preservation of cartilage homeostasis, regulation of subchondral bone remodeling, and, in selected studies, improvement of pain-related or functional outcomes. The principal preclinical in vivo studies evaluating MSC-EV-mediated immunomodulatory and joint-level effects in OA are summarized in Table 1.

### 2.4. Translational Development and Clinical Evidence for MSC-EVs in Osteoarthritis

#### 2.4.1. Translational Considerations for MSC-EV Development

Translation of MSC-EVs from experimental OA models to clinical therapy requires reproducible manufacturing, appropriate formulation, and sufficient IA persistence. Rapid clearance of EVs following IA administration may limit local exposure and therapeutic duration [143], motivating the development of sustained-release formulations and delivery systems designed to improve EV retention within the joint [93].

Manufacturing represents a second major translational challenge. Parental-cell culture conditions, isolation procedures, and formulation can influence EV composition, yield, purity, and biological activity, emphasizing the need for scalable and clinically compatible production methods. Translation to clinical-grade products additionally requires compliance with good manufacturing practice (GMP), including controlled and traceable starting materials, validated upstream and downstream processes, predefined release criteria, and demonstration of product identity, purity, sterility, potency, stability, and batch-to-batch consistency [144,145]. Chemically defined culture conditions have been used to generate AD-MSC-EVs with minimal detectable culture-medium contaminants while retaining biological activity in chondrocytes and experimental OA models [146,147]. Such approaches illustrate the importance of controlling upstream culture conditions and downstream processing when developing standardized EV products. Beyond manufacturing standardization, clinical development also presents regulatory challenges involving product classification, quality and potency assessment, and the establishment of appropriate safety and clinical evaluation frameworks [148,149].

Large-animal models can provide translational information that is not readily captured in rodent OA experiments because their joint dimensions, cartilage thickness, and biomechanical characteristics more closely approximate clinically relevant features of the human joint [150]. In a porcine osteochondral-defect model, IA administration of MSC-EVs in combination with hyaluronic acid (HA) promoted cartilage and subchondral bone repair compared with HA alone, without major adverse responses or systemic alterations during the study period [151]. Although osteochondral-defect models do not reproduce the full pathophysiology of OA, they can inform delivery feasibility, tissue integration, and safety in joints more comparable in scale to the human joint. In a separate minipig chondral-defect model incorporating bone-marrow stimulation, MSC-EV treatment increased subchondral bone ingrowth but was associated with impaired cartilage repair [152], indicating that EV-based interventions may interact with concurrent repair procedures and should be evaluated within the intended treatment context.

Together, these findings highlight that clinical translation will depend not only on biological potency but also on reproducible manufacturing, product characterization, local delivery and persistence, and evaluation in models that appropriately reflect the intended clinical application. Persistent heterogeneity in MSC source, culture conditions, EV isolation, characterization, and dosing across OA studies further emphasizes the need for standardized production and reporting frameworks [14].

#### 2.4.2. Clinical Evidence in Osteoarthritis

Clinical evaluation of MSC-EVs in OA remains substantially less developed than the preclinical literature. Available evidence is limited and heterogeneous, ranging from uncontrolled pilot studies and individual cases to small randomized clinical investigations, and therefore remains insufficient to establish therapeutic efficacy [153].

The most rigorous controlled evidence to date comes from a randomized, triple-blind, placebo-controlled trial in patients with bilateral Kellgren–Lawrence (KL) grade 2–3 knee OA. Each participant’s knees were randomized to receive a single IA injection of placental MSC-EVs or saline. No severe local reactions or systemic complications were observed; however, EV treatment did not produce significant improvements over placebo in visual analog scale (VAS), Western Ontario and McMaster Universities Osteoarthritis Index (WOMAC), Lequesne index, or magnetic resonance imaging (MRI) outcomes at 2 or 6 months [15]. These findings provide preliminary evidence of short-term tolerability but indicate that the therapeutic effects observed in preclinical models have not yet been consistently reproduced in controlled clinical settings.

A prospective open-label pilot study evaluated an IA BMSC-derived EV isolate product in patients with musculoskeletal OA [154]. A single 2-mL injection was administered to affected joints, with follow-up extending to one year. No treatment-related adverse events or clinical deterioration were reported, and improvements were observed across several patient-reported pain, disability, functional, and quality-of-life measures. Although these findings provide preliminary support for the feasibility of IA EV administration, the non-randomized design, heterogeneous joints treated, and absence of a control group limit conclusions regarding efficacy.

Evidence specifically using characterized MSC-derived EV preparations remains particularly sparse. A recent first-in-human report described IA administration of clinical-grade UC-MSC-EVs to a patient with symptomatic knee OA. Improvements in VAS and WOMAC scores were maintained at 6 and 12 months, together with improved walking capacity, with only transient post-injection pain and no persistent local or systemic adverse events reported during one year of follow-up [16]. As a single-patient report, however, this study establishes feasibility rather than treatment efficacy and requires confirmation in controlled trials.

A subsequent dose-ranging clinical study evaluated repeated IA administration of UC-MSC-derived exosomes in 41 patients involving 45 knees with mild-to-moderate OA. Participants were randomized among three dose groups receiving 3 × 10^11^, 4 × 10^11^, or 5 × 10^11^ particles per injection on days 0, 21, and 42. No significant safety differences or efficacy differences were observed among dose groups, although WOMAC scores improved within groups, particularly at the highest dose. MRI assessment in a subset of participants suggested reductions in bone-marrow edema and joint effusion and possible cartilage thickening [17]. Because the study lacked a placebo or untreated control group and MRI follow-up was incomplete, these findings provide preliminary safety and dose-ranging information rather than confirmatory evidence of efficacy. Moreover, although repeated administration was not associated with an apparent safety signal, dedicated assessment of alloimmune sensitization was not reported. This remains an important consideration for allogeneic MSC-EVs, as immune responses following repeated exposure remain insufficiently characterized [155,156].

Clinical studies using broader MSC secretome preparations provide additional but indirect evidence for cell-free MSC therapy. In a randomized study comparing IA UC-MSC secretome with HA, both groups improved in VAS and WOMAC scores over 12 weeks, with greater improvement reported in the secretome-treated group [157]. Because the administered product contained the broader MSC secretome rather than purified EVs, these findings cannot be attributed specifically to the EV fraction and are therefore more appropriately interpreted as supportive evidence for cell-free MSC-derived products.

By contrast, clinical studies administering intact MSCs should not be considered direct clinical evidence for EV therapy. Trials of IA UC-MSC administration have reported improvements in pain and function and changes in joint-associated biomarkers [158,159], but these interventions contain viable cells and therefore differ fundamentally from purified EV or secretome products. They may provide contextual evidence for the paracrine therapeutic rationale but should remain clearly distinguished from clinical evaluation of MSC-EVs.

Prospective clinical evaluation of MSC-EVs in knee OA is also beginning to expand. ClinicalTrials.gov currently includes a Phase I study of a single IA dose of allogeneic MSC-derived EVs (NCT05060107), an early Phase I dose-escalation study of UC-MSC-EVs (NCT06431152), and an open-label study evaluating repeated IA administration of allogeneic MSC-derived exosomes (NCT06466850). These studies remain small and are primarily designed to assess safety, feasibility, dose selection, and preliminary clinical outcomes rather than confirmatory efficacy. Additional EV-based OA trials are registered, although some involve non-MSC-EV sources or do not clearly specify the parental-cell source. Published and registered human studies of MSC-EV-based therapies in OA are summarized in Table 2.

Overall, clinical translation of MSC-EVs for OA remains at an early stage. Current evidence supports the feasibility and preliminary short-term tolerability of IA administration in small patient populations, with follow-up generally limited to 6–12 months. However, long-term safety remains uncertain, and evidence for clinical efficacy remains inconclusive. The small number of participants, heterogeneous EV products and cell sources, inconsistent dosing regimens, limited controls, and variable clinical and structural outcome assessments preclude definitive conclusions. Importantly, the absence of superiority over placebo in the available controlled trial contrasts with improvements reported in uncontrolled studies, underscoring the need for appropriately powered randomized trials. Future clinical development will require standardized product characterization, dose and administration schedules, appropriate comparators, and reproducible structural and patient-reported outcomes before the therapeutic value of MSC-EVs in OA can be established.

### 2.5. MSC Secretome Versus Extracellular Vesicles: Comparative Evidence and Therapeutic Implications

MSC-EVs represent one component of the broader MSC secretome, which also contains soluble proteins, lipids, metabolites, and other bioactive factors. Among these are immunomodulatory mediators such as PGE2, TSG-6, TGF-β, and IDO-associated tryptophan metabolites, as discussed in Section 2.2. Moreover, extracellular miRNAs are not exclusive to EVs and can also be present in non-vesicular protein complexes, including those associated with Argonaute proteins, although their contribution to MSC-mediated therapeutic effects in OA remains unclear [161]. Consequently, demonstration of therapeutic activity by isolated EVs does not establish that they account for all paracrine effects of MSCs. Determining whether EVs are sufficient to reproduce the activity of the complete secretome—or whether EVs and soluble mediators exert complementary effects—is therefore important for defining the most appropriate cell-free MSC-derived therapeutic product for OA [162].

Direct comparisons among whole conditioned medium (CM), isolated EV fractions, and EV-depleted CM provide the most informative evidence for addressing this question. In this context, CM represents the unfractionated MSC-conditioned product containing both EV-associated and soluble components, whereas EV-depleted CM provides an experimental approach for assessing the contribution of the non-vesicular fraction. Interpretation of these comparisons nevertheless requires caution because differences in EV isolation, normalization, concentration, culture conditions, and MSC source can result in preparations that are not quantitatively equivalent.

A comparative study evaluated BMSC-derived exosome- and microparticle (MP)-enriched fractions against BMSC-CM in inflammatory chondrocyte models and subsequently assessed the EV fractions alongside BMSCs in collagenase-induced OA. Both vesicular fractions restored anabolic–catabolic balance, exerted anti-apoptotic effects, suppressed macrophage activation, and protected against OA development in vivo, supporting the capacity of isolated EV fractions to reproduce major components of MSC paracrine activity [126]. These findings indicate that substantial therapeutic activity is retained after fractionation of the secretome, although they do not establish that soluble factors are dispensable.

Studies using AD-MSC-conditioned products further illustrate that the relative contribution of vesicular and soluble components may depend on the biological endpoint examined. Comparisons among CM, microvesicles (MVs), and exosomes in human OA osteoblasts, chondrocytes, and cartilage explants showed anti-inflammatory and anti-senescent activity across all preparations, although their relative effects differed by outcome. CM produced stronger suppression of TNF-α in osteoblasts, whereas MVs more effectively restored IL-10, collagen II, and Annexin A1 in selected systems. Proteomic analyses further demonstrated both shared and differentially enriched proteins among CM and EV fractions [163,164]. These observations argue against a simple model in which one secretome fraction uniformly accounts for all biological activity.

Additional comparative evidence indicates that AD-MSC CM may exert greater chondroprotective activity than isolated EVs when both preparations are derived from the same number of parental cells. Only CM significantly reduced MMP activity and PGE2 following inflammatory stimulation, although both CM and EVs reduced COL10A1 expression [165]. Importantly, the EV preparation contained substantially less total protein and fewer vesicles than the corresponding CM, illustrating a major challenge in interpreting head-to-head comparisons: apparent differences in potency may reflect both biological composition and differences in the amount of material delivered. Proteomic analyses also identified distinct enrichment of matrix-associated proteins between the two fractions.

Studies incorporating an EV-depleted condition provide additional evidence for vesicle-associated activity, although incomplete EV depletion and potential co-removal of non-vesicular components limit attribution of the observed effects exclusively to EVs. In embryonic stem cell-derived MSC (ESC-MSCs) preparations, EV-containing CM and isolated exosomes restored collagen II and reduced ADAMTS5 in inflammatory chondrocytes, whereas exosome-depleted CM showed substantially weaker effects. Isolated exosomes also attenuated cartilage degradation in vivo [166]. These findings support an important contribution of EVs to matrix-protective activity within the MSC secretome.

However, the presence of EVs does not necessarily imply that the soluble fraction is biologically irrelevant. In BMSC-derived preparations, EV-containing CM produced the greatest suppression of cyclooxygenase-2 (COX-2), whereas both EV-containing CM and isolated EVs enhanced proteoglycan production more effectively than EV-depleted CM [167]. This pattern is compatible with complementary activity between vesicular and soluble components rather than complete functional redundancy. In a separate comparison, isolated BMSC-EVs produced a strong early suppression of IL-6, IL-8, and COX-2, whereas CM produced a more sustained response at later time points [168]. Culture conditions also influenced both CM activity and EV production, further demonstrating that comparisons between cell-free products depend on how the parental MSCs are cultured and the resulting products are generated.

Comparisons of EVs with intact MSCs provide a related but distinct perspective. UC-MSCs and their derived small EVs produced comparable cartilage-protective and macrophage-associated effects in an OA model, including preservation of type II collagen and reduction in MMP13 and ADAMTS5. Proteomic analysis of the EV fraction identified cargo associated with immune regulation, matrix organization, and PI3K–AKT signaling [169]. These findings support the capacity of MSC-EVs to reproduce selected effects of their parental cells, but they do not resolve the relative contribution of EVs and soluble secretome components because an EV-depleted fraction was not evaluated.

Taken together, the comparative literature supports a major, but not necessarily exclusive, contribution of EVs to MSC secretome-mediated activity in OA. Isolated EVs frequently reproduce chondroprotective and immunomodulatory effects observed with unfractionated CM, and studies incorporating EV-depleted CM generally show loss or attenuation of important matrix-protective and anti-inflammatory responses. At the same time, several experiments demonstrate stronger or more sustained effects with unfractionated CM, indicating that soluble mediators may complement EV-mediated signaling. The principal direct comparisons of MSC-derived EVs, complete CM, and EV-depleted secretome in OA models are summarized in Table 3.

Accordingly, the current evidence does not support a universal conclusion that either isolated EVs or the complete secretome is intrinsically superior. EV preparations may offer a more defined product that can be concentrated, characterized, and modified independently of soluble secretome components, whereas unfractionated secretome may preserve potentially beneficial interactions between vesicular and soluble mediators. Determining which strategy is preferable for OA will require head-to-head studies using standardized parental-cell sources, culture conditions, EV isolation methods, dose-normalization strategies, and biologically relevant outcome measures. Such comparisons will be particularly important for distinguishing true differences in therapeutic mechanism from differences arising from product composition and experimental design.

## 3. Discussion and Future Perspectives

MSC-EVs have emerged as promising cell-free candidates for immunomodulation and joint protection in OA. Preclinical evidence most consistently supports modulation of macrophage-associated inflammatory responses, accompanied by reductions in synovitis, cartilage degeneration, subchondral bone alterations, and, in selected studies, pain-related outcomes. However, these associations do not necessarily establish that macrophage regulation is the principal mechanism responsible for joint protection. The contribution of specific immune pathways remains incompletely defined, particularly given the heterogeneity of MSC sources, EV preparations, OA models, and treatment regimens. Furthermore, OA-specific evidence for adaptive immune modulation remains comparatively limited, with much of the mechanistic understanding derived from non-OA inflammatory conditions. Establishing causal relationships between immune regulation and structural or functional improvements will therefore be essential for determining the therapeutic relevance of individual MSC-EV-mediated mechanisms.

Comparative studies further indicate that EVs are important but not exclusive contributors to MSC paracrine activity, suggesting that vesicular and soluble mediators may exert complementary effects. Although isolated EVs can reproduce several immunomodulatory and chondroprotective responses observed with the complete MSC secretome, differences in experimental normalization, EV recovery, and product composition complicate direct comparisons. Consequently, current evidence does not establish the universal superiority of isolated EVs or unfractionated secretome preparations. Further studies using matched MSC sources, standardized dosing, and appropriately characterized EV-containing and EV-depleted fractions are needed to clarify their relative contributions and identify the most suitable cell-free therapeutic strategies for OA.

Clinical translation remains at an early stage, with an important discrepancy between encouraging preclinical outcomes and the limited evidence of efficacy in humans. Although initial clinical investigations provide preliminary support for the feasibility and short-term tolerability of IA MSC-EV administration, therapeutic efficacy remains unestablished, including a lack of significant benefit over placebo in the available controlled trial [14]. These findings highlight the need to consider how differences between experimental OA models and heterogeneous human disease may influence therapeutic responses. Future development will require standardized MSC sourcing and culture conditions, rigorous EV characterization and potency testing, dose and delivery optimization, and adequately powered randomized clinical trials incorporating clinically meaningful structural and patient-reported outcomes. Importantly, identifying OA phenotypes most likely to respond to MSC-EV-mediated immunomodulation, while establishing whether changes in immune activity translate into sustained joint protection, will be critical for determining their clinical therapeutic value.

## 4. Methods

This narrative review was developed to critically examine the immunomodulatory potential of MSC-EVs in OA, with emphasis on underlying biological mechanisms, joint-level therapeutic responses, and clinical translation. Relevant literature was primarily identified through PubMed and Scopus using an iterative, topic-driven search approach involving keywords and combinations of terms related to OA pathophysiology, MSC paracrine signaling, extracellular vesicle biology, immune regulation, preclinical evidence, and clinical applications. Literature exploration was guided by the scientific questions addressed in the review and evolved as additional findings, mechanistic relationships, and knowledge gaps emerged. Publicly available clinical trial records from ClinicalTrials.gov were also consulted to identify registered studies investigating MSC-EV-based therapies for OA. The literature search was last updated in September 2026.

The literature was synthesized qualitatively, incorporating findings from experimental, preclinical, and clinical investigations alongside relevant reviews and methodological guidance. Particular attention was given to distinguishing OA-specific evidence from findings extrapolated from other inflammatory conditions, examining the consistency of reported biological effects, and identifying limitations associated with experimental models, EV preparations, and study designs. The manuscript was organized thematically, with individual sections developed and refined through continued examination and critical interpretation of the literature. As this was a narrative rather than a systematic review, no predefined systematic search protocol, formal study-screening procedure, or quantitative evidence synthesis was performed.

## Figures and Tables

**Table 1:** Preclinical in vivo studies of MSC-EV-mediated immunomodulation and joint protection in osteoarthritis.

MSC Source	EV Modification	OA Model	Treatment Design	Dose	Immunomodulatory/Mechanistic Effects	Joint/Functional Outcomes
**BMSC [12]**	None	Modified Hulth surgical OA (rat)	IA administration beginning 4 weeks after surgery; injections on 3 consecutive days per week for 4 weeks	1 × 10^8^ particles/injection in 10 µL	Reduced synovial macrophage infiltration and inflammatory macrophage-associated markers; increased ARG1-associated responses; reduced IL-1β, IL-6, and TNF-α and increased IL-10 in synovial fluid	Reduced cartilage damage and osteophyte formation; attenuated OA progression
**UC-MSC [97]**	None	ACLT-induced OA (rat)	Weekly IA administration	Not clearly reported for in vivo treatment	Increased ARG1/CD206-associated macrophage responses and IL-10; reduced TNF-α, IL-1β, and IL-6; PI3K–Akt signaling implicated in EV-mediated immunoregulation	Reduced cartilage degeneration and MMP13-associated catabolic changes
**AD-MSC [10]**	None	MIA-induced OA (rat)	IA administration on 3 consecutive days beginning 3 days after MIA induction	100 µg/injection in 100 µL	Reduced synovial and IFP inflammatory responses; single-cell transcriptomic analysis demonstrated altered macrophage states and inflammatory/joint-homeostatic signaling	Attenuated cartilage degeneration; improvement in selected pain- and gait-related measures
**IFP-MSC [98]**	None	MIA-induced OA (rat; acute synovial/IFP inflammatory phenotype)	Single IA injection 4 days after MIA induction; assessment 4 days after treatment	EVs corresponding to secretion from 5 × 10^5^ or 5 × 10^6^ IFP-MSCs in 50 µL	Reduced inflammatory macrophage-associated responses; increased CD206-associated relative to CD86-associated macrophage responses; reduced synovitis and IFP fibrosis	Reduced synovial inflammation and fibrosis
**IFP-MSC [11]**	None	ACLT-induced OA (mouse)	Single IA injection 1 week after surgery	3 µg in 7 µL	Reduced inflammatory macrophage-associated responses and expression of IL-1β, IL-6, and TNF-α	Preserved type II collagen and proteoglycans; reduced MMP13 and ADAMTS5 expression, osteophyte formation, and subchondral bone loss; improved gait-related outcomes
**SMSC [99]**	None; miR-3614-5p investigated mechanistically	ACLT-induced OA (rat)	IA administration	NR	Exosomal miR-3614-5p targeted ANXA2 and suppressed TLR4/MyD88/NF-κB signaling; reduced inflammatory macrophage infiltration	Reduced synovial inflammation, chondrocyte/ECM injury, and cartilage damage
**MSC [100]**	IL-6 priming	ACLT + DMM-induced OA (rat)	IA administration once weekly beginning 4 weeks after surgery	3 × 10^8^ particles/injection in 30 µL	Reprogrammed macrophage metabolism and phenotype through the mt-ND3/NADH-CoQ axis; IL-6-primed EVs showed greater immunomodulatory activity than unprimed EVs	Reduced synovitis and cartilage erosion
**BMSC [101]**	None	ACLT + medial meniscus resection-induced OA (rat)	Single IA injection 4 weeks after surgery	100 µg in 50 µL	Inhibited PINK1/Parkin signaling, mitochondrial damage, and inflammatory macrophage-associated responses; increased reparative macrophage-associated markers and IL-10 while reducing IL-1β, IL-6, and TNF-α	Reduced cartilage damage and OA progression
**SMSC [102]**	BMP-7 modification	MIA-induced OA (rat)	IA administration every 3 days beginning 3 weeks after MIA induction; assessment at week 8	1 × 10^10^ particles/injection in 100 µL	BMP-7-modified EVs enhanced CD206-associated macrophage responses and reduced iNOS-associated responses and inflammatory cytokines compared with unmodified EVs	Reduced cartilage injury and preserved cartilage matrix components
**iPSC-MSC [124]**	No direct EV cargo modification; serum-containing versus xeno-free production conditions and hydrogel delivery compared	DMM-induced OA (mouse)	Single IA injection 21 days after surgery, with or without hydrogel	1 × 10^7^ particles in 5 µL	Demonstrated retention of therapeutic activity after xeno-free EV production; hydrogel delivery was evaluated for local retention	Reduced joint damage and MMP13 expression; preserved type II collagen; attenuated subchondral bone sclerosis and normalized trabecular architecture
**WJ-MSC [125]**	None	ACLT-induced OA (rat)	IA administration once weekly for 4 weeks	30 µg/injection in 200 µL	EV miRNA profiles implicated calcium signaling, ECM–receptor interaction, and NOTCH pathways; let-7e-5p was identified as a candidate regulator of STAT3 and IGF1R	Promoted chondrogenic responses and protected cartilage and subchondral bone
**BMSC [126]**	None; exosomes compared with microparticles (MPs)	Collagenase-induced OA (mouse)	Single IA injection 7 days after OA induction	250 ng in 5 µL	Exosomes reduced inflammatory and catabolic responses and macrophage activation in complementary in vitro experiments	Preserved cartilage volume and thickness; reduced cartilage degradation and osteophyte formation; improved subchondral and epiphyseal bone parameters
**BMSC [127]**	None; lncRNA MEG3 investigated mechanistically	ACLT + DMM-induced OA (rat)	IA administration once weekly after surgery through week 8	100 µg/injection in 100 µL	Exosomal lncRNA MEG3 was implicated in reduced chondrocyte senescence and apoptosis	Attenuated cartilage destruction and abnormal subchondral bone remodeling; restored trabecular bone volume fraction, trabecular number, and connectivity
**BMSC [128]**	None	MIA-induced OA (rat)	Weekly IA administration	NR	Reduced IL-1β, TNF-α, and IL-6 expression and NF-κB p65 phosphorylation; promoted chondrocyte proliferation and reduced apoptosis	Reduced cartilage erosion, synovial hyperplasia, and inflammatory infiltration; restored type II collagen and aggrecan; improved joint function
**AD-MSC [129]**	None	MIA-induced OA (rat)	IA administration beginning 3 days after MIA induction, once weekly for 3 weeks	0.5 × 10^8^ or 1.0 × 10^8^ particles/injection in 50 µL	Reduced inflammatory and pain-associated signaling; complementary chondrocyte experiments implicated suppression of PI3K/Akt-associated inflammatory responses	Preserved type II collagen and joint integrity; improved paw-withdrawal latency, paw-withdrawal threshold, and weight-bearing performance
**IFP-MSC [130]**	None; endogenous miR-100-5p identified as active cargo	DMM-induced OA (mouse)	IA administration twice weekly beginning 4 weeks after surgery	1 × 10^8^ particles/injection in 10 µL	miR-100-5p inhibited mTOR signaling, enhanced autophagy, and reduced chondrocyte apoptosis and catabolic responses	Reduced cartilage degeneration and OARSI scores; preserved type II collagen; reduced MMP13 and ADAMTS5; partially improved gait abnormalities
**BMSC [131]**	miR-3960 enrichment	Surgically induced medial collateral ligament/medial meniscus injury OA (mouse)	IA administration once weekly for 3 weeks beginning 4 weeks after OA induction	1 µg/injection in 10 µL	EV-delivered miR-3960 targeted PHLDA2 and suppressed the SDC1/Wnt/β-catenin axis; reduced inflammatory signaling and chondrocyte apoptosis	Preserved type II collagen and aggrecan; reduced MMP13 and ADAMTS5 expression and cartilage injury
**MSC [132]**	circHIPK3 overexpression in parental MSCs	Collagenase-induced OA (mouse)	Single IA administration	NR	circHIPK3/miR-124-3p/MYH9 signaling promoted chondrocyte proliferation and reduced apoptosis	Reduced cartilage degradation and Mankin scores; increased aggrecan, COL2A1, and SOX9 and reduced RUNX2 and MMP13
**IFP-MSC [133]**	Wogonin pretreatment of parental MSCs	DMM-induced OA (mouse)	IA administration twice weekly from weeks 4 to 8 after surgery	1 × 10^10^ particles/injection in 10 µL	Wogonin-conditioned EVs increased SOX9, aggrecan, and type II collagen and reduced MMP13 and ADAMTS5 compared with unconditioned EVs	Reduced cartilage degeneration and OARSI/Mankin scores; wogonin-conditioned EVs demonstrated greater chondroprotective activity than unconditioned EVs
**SMSC [134]**	miR-140-5p overexpression	ACLT with medial collateral ligament and medial meniscus transection-induced OA (rat)	IA administration once weekly from weeks 5 to 8 after surgery	1 × 10^10^ particles/injection in 100 µL	miR-140-5p-enriched EVs promoted chondrocyte proliferation and migration and supported cartilage-homeostatic signaling	Reduced OA progression and OARSI scores; preserved chondrocyte number, aggrecan, and type II collagen and reduced type I collagen deposition
**iPSC-MSC and SMSC [135]**	None; MSC-source comparison	Collagenase-induced OA (mouse)	IA administration on days 7, 14, and 21 after OA induction	8 × 10^7^ particles/injection in 8 µL	Both EV preparations promoted chondrocyte proliferation and migration, with source-dependent differences in biological activity	Both preparations reduced OA severity and preserved cartilage morphology, proteoglycans, and type II collagen; iPSC-MSC-EVs produced greater cartilage protection
**IFP-MSC [136]**	CD10^High^EV preparation	MIA-induced OA (rat; acute synovial/IFP inflammatory phenotype)	Single IA injection 4 days after MIA induction; assessment 4 days after treatment	CD10^High^EVs in 50 µL; preparation corresponding to EVs secreted by approximately 1 × 10^6^ IFP-MSCs	CD10^High^EVs displayed immunomodulatory miRNA cargo and chondroprotective activity; preservation of PRG4-associated cartilage homeostasis	Reduced cartilage degeneration; increased collagen composition; preserved superficial-zone PRG4 expression
**AD-MSC (subcutaneous adipose tissue) [137]**	miR-199a-3p loading; chondrocyte-targeting CAP–Lamp2b modification	DMM + ACLT-induced OA (rat); DMM-induced OA (mouse, engineered-EV experiments)	Rats: IA once weekly for 6 weeks beginning 3 weeks after surgery; mice: IA once weekly for 4 weeks beginning 6 weeks after surgery	Rats: 1 × 10^9^ particles/injection in 50 µL; mice: 1 × 10^8^ particles/injection in 10 µL	EV-delivered miR-199a-3p inhibited mTOR signaling and enhanced autophagy; miR-199a-3p antagonism attenuated EV-mediated cartilage protection; CAP modification enhanced cartilage targeting	Reduced cartilage degeneration and OARSI scores; increased COL2A1 and reduced MMP13; miR-199a-3p-loaded and CAP-targeted EVs produced enhanced cartilage protection
**DP-MSC [138]**	None	MIA-induced OA (mouse)	Single IA injection 2 weeks after MIA induction	2 × 10^8^ particles in 10 µL	Suppressed TRPV4-associated osteoclast activation and inflammatory responses	Improved subchondral bone remodeling; reduced bone sclerosis, osteophyte formation, cartilage degradation, and synovial inflammation
**BMSC [140]**	TGF-β1 pretreatment of parental BMSCs; EVs enriched in miR-135b	ACLT-induced OA (mouse)	IA administration after ACLT	1 × 10^9^ particles/injection in 10 µL	Reduced osteoclastogenesis and PDGF-BB-associated CD31^hi^Emcn^hi^ H-type vessel activity; complementary mechanistic studies implicated MAPK inhibition	Preserved cartilage and subchondral bone microarchitecture; reduced pathological angiogenesis and OA-associated pain behavior
**IFP-MSC [141]**	AAV-mediated CGRP_8−37_ antagonist expression in parental IFP-MSCs	MIA-induced OA (rat; acute synovial/IFP inflammatory phenotype)	Single IA injection 4 days after MIA induction; behavioral assessment through day 13 after EV treatment and histological evaluation at day 30 after MIA	100 µL at 3–4 × 10^9^ EVs/mL (~3–4 × 10^8^ EVs/injection)	EVs carried CGRP antagonist and CD10/neprilysin-related activity; complementary experiments demonstrated reduced neuroinflammatory signaling and cartilage-homeostatic responses	Reduced tactile and cold allodynia and knee-bend pain; preserved cartilage structure and collagen organization; reduced matrix degradation and preserved PRG4-associated localization

**Abbreviations:** ACLT, anterior cruciate ligament transection; AD-MSC, adipose-derived mesenchymal stem/stromal cell; BMSC, bone marrow mesenchymal stem/stromal cell; DMM, destabilization of the medial meniscus; DP-MSC, dental pulp mesenchymal stem/stromal cell; ECM, extracellular matrix; EV, extracellular vesicle; IA, intra-articular; IFP-MSC, infrapatellar fat pad mesenchymal stem/stromal cell; iPSC, induced pluripotent stem cell; lncRNA, long noncoding RNA; MIA, monosodium iodoacetate; MSC, mesenchymal stem/stromal cell; MPs, microparticles; NR, not reported or not sufficiently specified in the primary report; OA, osteoarthritis; SMSC, synovial mesenchymal stem/stromal cell; UC-MSC, umbilical cord mesenchymal stem/stromal cell; WJ-MSC, Wharton’s jelly mesenchymal stem/stromal cell. **Note:** Mechanistic effects include pathways investigated in complementary in vitro experiments and/or supported by in vivo molecular analyses; they should not be interpreted as having been demonstrated exclusively in vivo in every study.

**Table 2:** Published and registered human studies of MSC-derived extracellular vesicle therapies in osteoarthritis.

Study/MSC-EV Product	Study Design and Population	Treatment	Follow-Up/Outcomes	Main Findings/Status
**Placental MSC-EVs [15]**	Randomized, triple-blind, placebo-controlled; 31 patients with bilateral KL 2–3 knee OA; 29 analyzed	Single IA injection; 5 mL at 7 × 10^9^ particles/mL; contralateral knee received saline	6 months; VAS, WOMAC, Lequesne index, MRI	No severe local or systemic complications; no significant difference from saline in clinical or MRI outcomes
**BMSC-derived EV isolate product [154]**	Prospective, open-label, uncontrolled pilot; 33 patients with OA involving multiple joints	Single 2-mL IA injection	12 months; pain, disability, function, quality of life, safety	Improvements in several patient-reported outcomes; no treatment-related deterioration reported; efficacy limited by uncontrolled design and heterogeneous joints
**Clinical-grade UC-MSC-EVs [16]**	First-in-human single-patient report; symptomatic knee OA	Single IA injection; approximately 2 × 10^10^ particles	12 months; VAS, WOMAC, walking capacity, safety; MRI	Sustained improvements in VAS and WOMAC and improved walking capacity; transient post-injection pain; no persistent local or systemic adverse events
**UC-MSC-derived exosomes [17]**	Randomized, double-blind dose-ranging study; 41 patients/45 knees, KL 2–3 OA; no placebo group	3 × 10^11^, 4 × 10^11^, or 5 × 10^11^ particles; IA injections on days 0, 21, and 42	9 months; WOMAC, safety laboratory measures; MRI in subset	No significant between-dose efficacy or safety differences; within-group WOMAC improvements reported; MRI subset suggested reduced edema/effusion and possible cartilage thickening; efficacy remains uncontrolled
**Allogeneic UC-MSC-EVs (NCT05060107)**	Phase I, open-label, single-arm; planned n = 10, KL 2–3 knee OA	Single IA dose; 3–5 × 10^11^ particles	12 months; safety, VAS, WOMAC, synovitis, OMERACT-OARSI response	Status: Unknown; no results posted
**Allogeneic UC-MSC-EVs (NCT06431152)**	Early Phase I, open-label dose escalation; planned n = 12, KL 2–3 knee OA	Single IA dose; 2 × 10^9^, 6 × 10^9^, or 2 × 10^10^ particles	12 months; safety, VAS, WOMAC, dose selection	Status: Unknown; last known status: recruiting; no results posted
**Allogeneic MSC-derived exosomes (tissue source not reported—NCT06466850)**	Open-label, single-group study; planned n = 20, knee OA	Repeated IA administration on days 1 and 90; particle dose not reported in registry	6 months; WOMAC and clinical outcomes	Status: Recruiting; no results posted

**Abbreviations:** BMSC, bone marrow-derived mesenchymal stem/stromal cell; EV, extracellular vesicle; HA, hyaluronic acid; IA, intra-articular; KL, Kellgren–Lawrence; MRI, magnetic resonance imaging; MSC, mesenchymal stem/stromal cell; OA, osteoarthritis; OMERACT-OARSI, Outcome Measures in Rheumatology–Osteoarthritis Research Society International; UC-MSC, umbilical cord-derived mesenchymal stem/stromal cell; VAS, visual analog scale; WOMAC, Western Ontario and McMaster Universities Osteoarthritis Index. **Note:** ClinicalTrials.gov records were last accessed on 30 September 2026. The MSC tissue source for NCT05060107 was identified from an investigator-reported conference abstract [160], whereas the tissue source for NCT06466850 was not specified in the available trial registration.

**Table 3:** Direct comparative studies of MSC-derived EVs, conditioned medium, and EV-depleted secretome in osteoarthritis models.

MSC Source	Experimental Model	Products Compared	Normalization/Comparability	EV Effect	Secretome/Soluble-Fraction Effect	Interpretation
**BMSC** [126]	In vitro: IL-1β-stimulated murine OA-like chondrocytes; macrophage activation assays. In vivo: collagenase-induced OA (mouse)	CM vs. exosome-enriched fraction vs. MP-enriched fraction in vitro; BMSCs vs. exosomes vs. MPs in vivo	EV fractions normalized by total protein; in vitro CM and isolated EV fractions were not quantitatively matched. No EV-depleted CM was tested. In vivo EV doses corresponded to the 48-h output of 2.5 × 10^5^ BMSCs	Exosomes and MPs restored anabolic–catabolic balance, reduced apoptosis and macrophage activation, and protected cartilage and bone in vivo	CM exerted chondroprotective effects in the same in vitro model; soluble-only activity was not directly tested	EV fractions reproduced major components of BMSC paracrine activity, but the absence of an EV-depleted CM condition prevents determining whether soluble mediators are dispensable
**AD-MSC** [163]	In vitro: IL-1β-stimulated human OA chondrocytes and cartilage explants	CM vs. MVs vs. exosomes	MV and exosome concentrations were selected to approximate those present in the CM used in the same experiments; no EV-depleted CM	Both EV fractions reduced inflammatory and catabolic mediators and preserved cartilage-associated responses; MVs were particularly effective in increasing IL-10, collagen II, and Annexin A1	CM produced broadly similar anti-inflammatory/chondroprotective effects, but relative potency differed by endpoint	EVs reproduced much of CM activity, with some endpoints favoring MVs; however, absence of a soluble-only fraction prevents attribution of residual CM activity specifically to soluble mediators
**AD-MSC** [164]	In vitro: IL-1β-stimulated human OA osteoblasts	CM vs. MVs vs. exosomes	MV and exosome concentrations were within the range present in the corresponding CM; no EV-depleted CM	MVs and exosomes reduced senescence, IL-6, PGE2, oxidative stress, and mitochondrial dysfunction and increased IL-10	CM produced broadly similar effects but, unlike MVs or exosomes, significantly reduced TNF-α	EVs reproduced many paracrine effects, whereas the selective effect of CM on TNF-α supports a non-redundant contribution from soluble mediators for specific endpoints
**AD-MSC** [165]	In vitro: TNF-α-stimulated human OA chondrocytes	CM vs. isolated EVs	Preparations originated from the same number of parental cells, but ultracentrifugation recovered only ~30% of CM particles; CM therefore contained more particles and substantially more total protein than the isolated-EV preparation. Proteomics used equal protein input	EVs reduced COL10A1 but did not significantly suppress MMP activity or PGE2	CM reduced COL10A1 and significantly reduced MMP activity and PGE2; CM was enriched in chondroprotective proteins including TIMP-1/-2	CM showed greater activity for selected outcomes, but unequal recovery of particles and protein prevents concluding that soluble factors are intrinsically more potent; nevertheless, compositional differences support biologically distinct contributions
**ESC-MSC** [166]	In vitro: IL-1β-stimulated primary mouse chondrocytes; in vivo: DMM-induced OA (mouse) for isolated exosome efficacy	EV-containing CM vs. exosome-depleted CM vs. isolated exosomes	Fractions were generated from the same ESC-MSC culture; isolated exosomes were tested separately and dose-dependently. Exact equivalence between isolated exosome dose and CM-associated exosome content was not established	Isolated exosomes restored collagen II and reduced ADAMTS5; EVs also attenuated cartilage degradation in vivo	EV-containing CM restored collagen II and reduced ADAMTS5, whereas exosome-depleted CM had substantially weaker effects	Evidence supporting a substantial contribution of EV-associated components to matrix protection of the ESC-MSC secretome, although residual activity of EV-depleted CM indicates that the non-vesicular fraction is not necessarily inactive
**BMSC** [167]	In vitro: TNF-α-stimulated human OA chondrocytes and 3D cartilage-regeneration cultures	CM vs. EV-depleted CM vs. isolated EVs	All preparations were normalized to the secretory output of the same number of BMSCs (equivalent to 5 × 10^5^ parental cells), providing comparatively strong product matching	EVs reduced inflammatory signaling and promoted proteoglycan and type II collagen production	CM produced comparable matrix-promoting effects; EV depletion strongly reduced proteoglycan and collagen II responses. For some endpoints, including COX-2 suppression and proliferation, CM retained stronger activity	Supports a substantial contribution of EV-associated components to cartilage matrix responses, while soluble factors also influence inflammatory and proliferative outcomes. However, EV-depletion limitations prevent definitive attribution of these effects to EVs alone
**BMSC** [168]	In vitro: IL-1α-stimulated human OA chondrocytes; comparison of FBS- and xeno-free MSC culture conditions	Complete CM (CM+) vs. EV-depleted CM (CM−) vs. isolated EVs	Comparisons were normalized according to parental-cell number; EVs were tested at 1 μg/mL with corresponding CM+ and CM− preparations. Culture conditions substantially altered EV yield and secretome activity	EVs produced rapid suppression of IL-6, IL-8, and COX-2 at 16 h, but this effect was largely lost by 48 h	Xeno-free CM+ showed a later, more sustained anti-inflammatory response; EV-depleted xeno-free CM also retained activity in selected conditions	Supports temporal and functional complementarity between EVs and soluble mediators; also demonstrates that culture conditions can alter the apparent relative contribution of each fraction

**Abbreviations:** AD-MSC, adipose-derived mesenchymal stem/stromal cell; BMSC, bone marrow-derived mesenchymal stem/stromal cell; CM, conditioned medium; CM+, complete EV-containing conditioned medium; CM−, EV-depleted conditioned medium; DMM, destabilization of the medial meniscus; ESC-MSC, embryonic stem cell-derived mesenchymal stem/stromal cell; EV, extracellular vesicle; MP, microparticle; MV, microvesicle; OA, osteoarthritis.

## Data Availability

No original datasets were generated in this study. All information supporting this narrative review is available in the cited literature.

## Abbreviations

The following abbreviations are used in this manuscript:

ACLT	Anterior cruciate ligament transection
AD-MSC	Adipose-derived mesenchymal stem/stromal cell
BMSC	Bone marrow-derived mesenchymal stem/stromal cell
CM	Conditioned medium
COX-2	Cyclooxygenase-2
DC	Dendritic cell
DMM	Destabilization of the medial meniscus
DP-MSC	Dental pulp-derived mesenchymal stem/stromal cell
ECM	Extracellular matrix
ESC-MSC	Embryonic stem cell-derived mesenchymal stem/stromal cell
EV	Extracellular vesicle
FBS	Fetal bovine serum
GMP	Good manufacturing practice
HA	Hyaluronic acid
IA	Intra-articular
IDO	Indoleamine 2,3-dioxygenase
IFP-MSC	Infrapatellar fat pad-derived mesenchymal stem/stromal cell
IL	Interleukin
iPSC	Induced pluripotent stem cell
KL	Kellgren–Lawrence
lncRNA	Long non-coding RNA
MIA	Monosodium iodoacetate
miRNA	MicroRNA
MMP	Matrix metalloproteinase
MP	Microparticle
MRI	Magnetic resonance imaging
MSC	Mesenchymal stem/stromal cell
MSC-EV	Mesenchymal stem/stromal cell-derived extracellular vesicle
mTOR	Mechanistic target of rapamycin
MV	Microvesicle
NK	Natural killer
NR	Not reported
OA	Osteoarthritis
OMERACT-OARSI	Outcome Measures in Rheumatology–Osteoarthritis Research Society International
PGE2	Prostaglandin E2
PRG4	Proteoglycan 4
SMSC	Synovial mesenchymal stem/stromal cell
TGF-β	Transforming growth factor beta
TNF-α	Tumor necrosis factor alpha
TSG-6	Tumor necrosis factor-stimulated gene-6
UC-MSC	Umbilical cord-derived mesenchymal stem/stromal cell
VAS	Visual analog scale
WJ-MSC	Wharton’s jelly-derived mesenchymal stem/stromal cell
WOMAC	Western Ontario and McMaster Universities Osteoarthritis Index
